# Hourly potential light availability maps at 10 m resolution over Switzerland

**DOI:** 10.1038/s41597-025-06152-9

**Published:** 2025-11-28

**Authors:** Clare Webster, Christian Ginzler, Mauro Marty, Anita Nussbaumer, Giulia Mazzotti, Tobias Jonas

**Affiliations:** 1 WSL Institute for Snow and Avalanche Research SLF, Davos, Switzerland; 2 Department of Geosciences, University of Oslo, Oslo, Norway; 3 Department of Geography, University of Zurich, Zurich, Switzerland; 4 Swiss Federal Institute for Forest, Snow and Landscape Research WSL, Birmensdorf, Switzerland; 5 Univ. Grenoble Alpes, INRAE, CNRS, IRD, G-INP, IGE, Grenoble, France; 6 Laboratory of Hydraulics, Hydrology and Glaciology (VAW), ETH Zurich, Zurich, Switzerland

**Keywords:** Forest ecology, Forestry, Hydrology, Biodiversity, Energy modelling

## Abstract

This paper presents the SwissRad10 dataset, containing novel nationwide light availability maps at 10 m and hourly resolution across an entire annual solar cycle. The variables sky-view factor and direct-beam transmissivity are calculated, allowing direct estimation of both direct and diffuse light at the ground surface. The dataset is calculated using the detailed synthetic hemispheric image-based Canopy Radiation Model (CanRad) with the latest airborne lidar and terrain surface data from the swissSURFACE3D dataset and resolves the shadow cast by every single tree in Switzerland. Below and adjacent forest canopy pixels include light availability calculated for both leaf-on and leaf-off canopy structure, and all pixels in the domain include a terrain-only calculation. This data is important for, e.g. assessing forest microhabitat suitability for both plants and animals, for disciplines linked via forest microclimate such as biogeochemistry and snow modelling, as well as for ecosystem research targeting carbon and water fluxes. The nationwide light availability maps presented in this paper will offer valuable insights into the dynamics and drivers of light availability, serving as a valuable resource for researchers, practitioners, and decision-makers working in the field of ecology and environmental sciences.

## Background & Summary

The amount of solar radiation reaching the land surface, either on top, below or outside forest canopy, controls the resulting light availability, which plays an important role in governing almost all ecological and hydrological processes. Light availability at the land surface is therefore the primary metric that can be used to determine how much shortwave radiation energy and light is available for most ecological and hydrological surface processes^1–5^. The spatial and temporal variability in light availability directly controls important hydrological processes such as snowmelt and evapotranspiration, varying across open and forested landscapes at hourly to inter-annual timescales^6,7^. Ecological processes such as plant photosynthesis and microclimate buffering are also strongly controlled by light availability, impacting vegetation health and growth, habitat suitability and plant and animal behaviour^2,8^. In Switzerland, approximately 80% of the land area is classified as natural landscapes, either forests, alpine regions or other ecological systems. Understanding and predicting light availability at national scales is therefore crucial for hydrological and ecological research, as well as conservation planning and land management strategies.

Across Switzerland, both terrain topography and forests significantly alter light availability at the land surface through the casting of shadows at different times of day. Complex local topography can influence light availability at the scale of several metres, causing isolated areas to remain in shade all day. At the same time large topographic features, such as the Alps influence light availability over 20 km away^9,10^, meaning that light availability almost everywhere in Switzerland is influenced by the presence of mountains. At the same time, over 30% of Switzerland is covered by forests, which have both natural and human-induced structural heterogeneity that significantly alters light availability from minute to seasonal scales, compared to non-forested areas. Complex shadow patterns move across the landscape as a function of solar elevation and azimuth angles, the position of individual trees and the size, location and distribution of forest gaps. The changing foliage density of deciduous forests also means that the canopy structure is not static throughout the year, and seasonal variation in light availability is not just a function of solar position but also of leaf unfolding and shedding phenology. Mapping light availability across national scales for research and management applications must therefore include both high-resolution topographic information and detailed canopy structure information capable of representing the small-scale light patterns created by individual tree crowns.

The most efficient way to calculate light availability at the land surface is by using two key variables that describe the transmission of light through both the atmosphere and the canopy: (1) sky-view factor (SVF), which is commonly used as a proxy for diffuse transmissivity^4^ and (2) time-varying direct-beam transmissivity (DBT)^11^. SVF describes the portion of sky visible across the entire hemisphere from the perspective of the ground and corresponds to diffuse light availability^12^. SVF can be calculated outside forests, incorporating the effect of terrain shading. Inside forests, SVF is influenced by both terrain shading and local canopy structure. DBT describes the amount of direct sunlight reaching the land surface at any given time in relation to solar zenith and azimuth position and determines whether the land surface is sun-lit or in shadow. Outside forests, the DBT is dictated by the position of the sun above or below the terrain and can be used with a calculated atmospheric transmissivity to determine the amount of direct radiation reaching the land surface. Within forests, the DBT is the fraction of direct radiation that penetrates through the canopy without being intercepted by the leaves or branches. Calculating light availability with SVF and DBT together allows for spatially explicit, realistic and complete estimation of the total amount of light availability at the land surface. Importantly, SVF and DBT also capture both the variability and the quality of light with respect to canopy structure, providing the necessary detail for ecological and hydrological research as well as forest and land use management.

SVF and DBT can be accurately calculated from hemispheric photographs^13,14^, but manually acquired photographs are not suitable for calculating light availability across spatial extents such as Switzerland. Crucially, the calculation of synthetic hemispheric images from remotely sensed forest structure datasets now mean SVF and DBT can be calculated across the scales at which these data are available^15–18^ with the methods adaptable to different forest types. Models that seek to both understand and predict ecohydrological land surface processes are almost always dependent on these spatially and temporally explicit light availability variables. For example, in topographic downscaling of shortwave and longwave radiation^19^, snowmelt modelling^20,21^ and high resolution microclimate mapping^5^.

This paper presents the SwissRad10 dataset of SVF and DBT at 10 m across all of Switzerland, with average DBT at hourly temporal resolution across an annual solar cycle. The dataset is calculated utilising the swissSURFACE3D data^22^ within the Canopy Radiation Model (CanRad^18^). The swissSURFACE3D project has generated high-resolution 3D models across Switzerland using airborne lidar data to collect elevation and forest structure data. The high spatial resolution of these data means that terrain variability at metre-scales and every individual tree in Switzerland are represented. The Canopy Radiation Model has been specifically developed for the purposes of predicting light availability across large spatial scales but maintaining metre-scale spatial and minute-scale temporal model resolution. The model uses terrain data and a canopy height model (CHM) to generate synthetic hemispheric images, which are subsequently used to calculate SVF and DBT following the methods in^14^. Within forests, both leaf-on and leaf-off scenarios are calculated, and light availability in open areas and above canopy are also calculated, creating three different dataset layers: leaf-on, leaf-off and terrain-only. This dataset is, to our knowledge, the first national-scale light availability dataset that incorporates the effects of every individual tree at high temporal and spatial resolution. The dataset will be an important tool for landscape-scale ecological and hydrological modelling within Switzerland but could also be used as a test and calibration dataset for method development using coarser resolution data.

## Methods

### Domain

Switzerland covers 41,285 km^2^ in central Europe. 70% of the country is classified as mountainous, while the remaining 30% are lowlands with relatively low relief. Elevation across Switzerland ranges from around 200 m.a.s.l in the north-east, to steep peaks exceeding 4000 m.a.s.l in the southern and central Swiss Alps. 31% of the land area is covered by forest, comprising predominantly deciduous broadleaf forests in the lowland regions, and evergreen needleleaf forests in the alpine regions. Alpine forests extend up to 2400 m.a.s.l^23^, above which grasslands and scree slopes dominate. Additionally, about 6% of the country is covered by trees outside of forests^24^. Another 35% of the land cover is classified as agricultural, which includes arable land as well as mountain pastures.

### Nationwide datasets as model input

Datasets used in the calculation of SVF and DBT were required to have complete coverage over the entire Swiss model domain as well as areas outside Switzerland that influence light availability within the model domain. Swiss-wide datasets include those from swisstopo and other publicly available sources. Additionally, forest and terrain data from the Copernicus Land Monitoring Service (CLMS) were used to supplement non-Swiss terrain influences and complement the Swiss forest type datasets.

#### Canopy height model

**Fig. 1:**
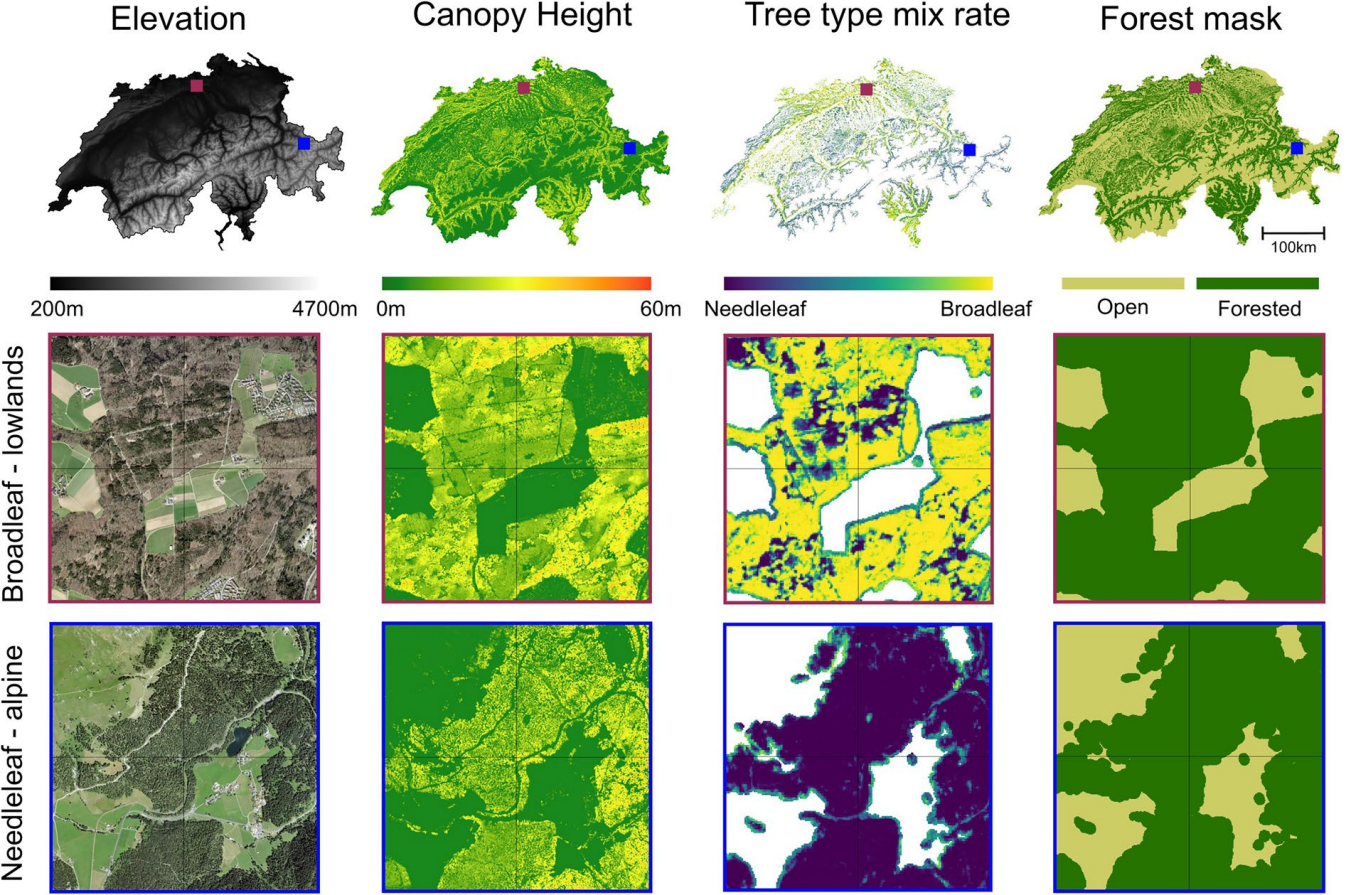
Overview and examples of datasets used in calculating light availability across Switzerland. Left to right: Elevation (5 m resolution), canopy height (1 m resolution), tree type mix rate (10 m resolution) and the forest mask (10 m resolution) are shown across Switzerland (top row). Examples of the forest structure are shown over a deciduous broadleaf forest from north Switzerland (middle row; red box), including a winter-time aerial image, canopy height, tree type and the forest mask. The same example is shown for an alpine needleleaf forest in east Switzerland (bottom row; blue box). Aerial image source: SWISSIMAGE 10 cm^44^.

A national CHM at 1 m resolution was calculated from airborne lidar data acquired from 2012–2023 (Fig. 1). The lidar data ranges in point density from 5–35 points per m^2^ and is primarily (82%) from the swissSURFACE3D dataset^22^, with supplementary data from cantonal acquisitions where the nationwide acquisition campaign had not yet been completed (18% of Switzerland; specifically, Bern^25^ 13%, Solothurn^26^ 2%, Basel Landschaft^27^ 2%, Rest 1%).

#### Forest data

We used three datasets to account for differences in forest type across the country, allowing for variable tree crown foliage densities, as well as distinguishing between deciduous and evergreen trees in mixed forests for calculating leaf-on and leaf-off light availability.

The forest type mix rate data is a 10 m resolution product that describes the proportion of deciduous and evergreen trees in each pixel^28^ (Fig. 1). Since few evergreen broadleaf species grow in Switzerland and only in isolated locations, all evergreen species were assumed to be coniferous. Additionally, larch trees (deciduous needleleaf) are included in the deciduous classification, but are structurally different from broadleaf deciduous trees included in the same category. We therefore used the Swiss forest ecoregions dataset^29^ to distinguish deciduous broadleaf and needleleaf forests. The dataset distinguishes different forest regions based on climatic factors, forest vegetation and altitudinal vegetation belts. We simplified this dataset to subdivide Switzerland into areas where the classification of deciduous in the mix rate data were likely to be broadleaf (e.g. the Swiss Plateau, Southern Pre-Alps) and where they were likely to be needleleaf (e.g. High Alps, Northern Central Alps). This simplification also distinguishes between evergreen forests across Switzerland, which are pine dominated in the Swiss Plateau but Norway spruce dominated in the alpine forest regions, allowing distinction when calculating the dataset.

Finally, we also included the 2018 10 m dominant leaf type (DLT) dataset from the Copernicus Land Monitoring Service^30^, which distinguishes between broadleaf and needleleaf forests. During visual inspection of the data, we noticed that in some locations above 1500 m.a.s.l, where the Swiss ecoregions dataset indicated needleleaf forests, larch forests were incorrectly classified as broadleaved forests in the DLT dataset, while at the same time the mix rate data had incorrectly classified the larch forests as evergreen (mix rate less than 50%). We leveraged this information to force the model to assume a larch forest, where all three of the above criteria were met. This correction was applied to only 1% of the total area of the leaf-off dataset but was considered valuable for forest snow modelling applications (e.g.^20^) when the distinction between alpine larch and spruce forests in winter is important.

#### Terrain

Two separate digital terrain models (DTMs) enabled us to represent both local (between point) and regional (topographic horizon line) terrain effects on radiation transfer. The local DTM utilised the swissALTI3D dataset^31^ at 5 m spatial resolution, which covers entire Switzerland, but is not available beyond the borders. The regional DTM was at 50 m spatial resolution. Calculation of the regional topographic horizon line included all terrain within a 25 km radius, thus requiring terrain information up to 25 km outside of the Swiss border. In regions within this radius where the local or regional DTMs contained no data, we used data from the Copernicus GLO-30 digital surface model^32^ and only the regional DTM was used to calculate the synthetic hemispheric images in these locations.

#### Forest cover mask

We used a forest mask to distinguish areas within Switzerland where light availability is influenced by the presence of forests. The final forest mask was calculated using (1) a forest mask calculated from the swissTLM3D (Topographic Landscape Model; based on aerial image interpretation^33^ and (2) the National Forest Inventory (NFI) mask based on stereo-imagery over Switzerland^34^. Both datasets were combined to ensure maximum probability of trees included in the forest mask. Additionally, a 25 m buffer was added around all forest edges. The forest mask was rasterised to 10 m resolution, creating the sampling points for the model run, where 0 = no model run (i.e. outside Switzerland); 1 = forest; 2 = open.

### Model description

**Fig. 2:**
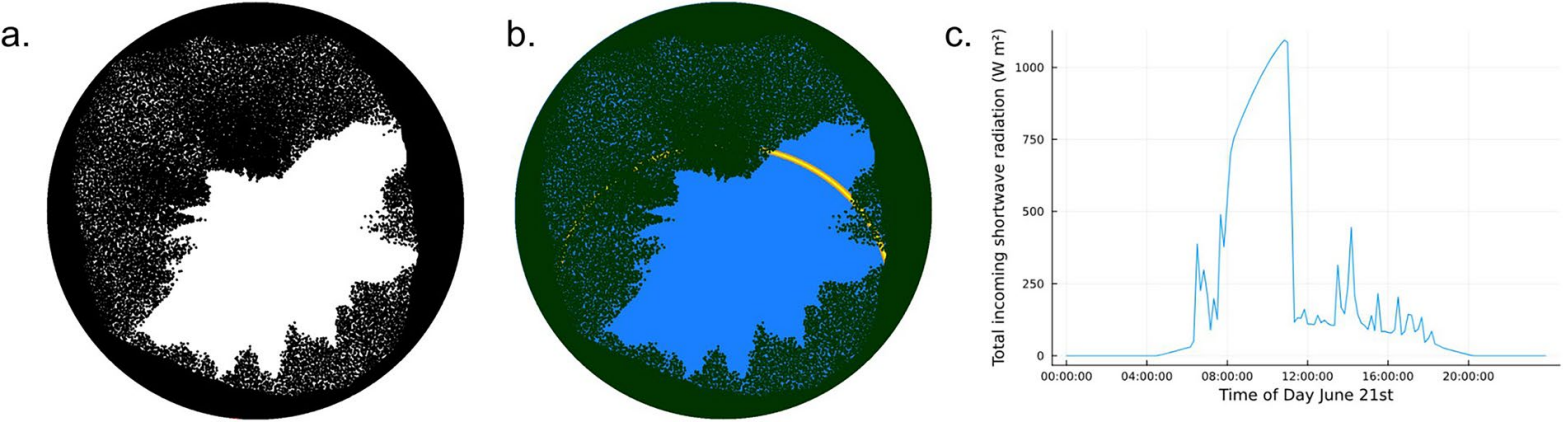
Example of a synthetic hemispheric image showing (**a**) sky-view factor as the ratio of white pixels in the full hemisphere, (**b**) daily variation of direct beam transmissivity on June 21st (yellow line) and (**c**) daily variation in maximum potential incoming shortwave radiation for the same day. This image was calculated in a 100% deciduous needleleaf forest in eastern Switzerland, using the same methods and data applied to the dataset calculation. Hemispherical images are oriented with south to top and east to right.

We used The Canopy Radiation Model (CanRad; https://github.com/c-webster/CanRad.jl), which calculates SVF and DBT from synthetic hemispheric images (Fig. 2), generated from digital surface models (terrain or canopy height).

CanRad consists of several sub-models. The model was originally developed to calculate synthetic hemispheric images and associated light availability from airborne lidar data (Lidar-to-Radiation (L2R)^17^) and is used as input to hyper-resolution snowmelt models^18,21,35^. expanded on these concepts to develop CanopyHeightModel-to-Radiation (C2R), using just a canopy height model to calculate the synthetic hemispheric images, with the goal of creating a detailed radiation transfer model suitable for input into large-scale forest energy balance calculations. Output from C2R has been used in forest microclimate mapping^5^, quantification of forest edge structures^36^, and national operational snowmelt modelling in Switzerland^20^. A third sub-model, Terrain-to-Radiation (T2R) is now included in CanRad and calculates synthetic hemispheric images of open terrain using one or two DTMs, based on the concepts already presented in^14,16,17^. Both C2R and T2R were used to calculate the SwissRad10 dataset.

T2R calculates the terrain horizon line using local and regional terrain models in two stages. In the first stage, the horizon line is calculated using the 5 m resolution DTM out to 300 m around each point. The smaller search radius and higher resolution DTM represents the local terrain differences between each model location. In the second stage, a horizon line is calculated from the 50 m DTM including topography within a 25 km radius. The two horizon lines are then combined to form one topographic horizon line and are used to create a synthetic hemispheric image. The horizon line is calculated at 1° azimuthal increments.

Within forests, C2R is used in addition to T2R to include the effect of forests on light availability. C2R uses information contained within a canopy height model to create synthetic hemispheric images based on the geometric arrangement of the surrounding canopy and applying a statistical correction for canopy transmissivity. A detailed model description and validation are described in^18^ but the model is briefly described here with respect to how it was applied over Switzerland.

C2R calculates the top-of-canopy horizon line from the 1 m CHM at 1° azimuthal increments while at the same time calculating canopy thickness along each azimuthal increment and 90 additional 1° zenith angle increments. C2R then applies a statistical correction for all regions of the hemispheric image below the canopy horizon line, calculating the probability of light penetrating the canopy as a function of path length and foliage area volume density of the canopy:

1 $${p}_{t}=\exp \left[-G\left(\theta \right)\sum \lambda {l}_{t}\right]$$

where G is the projection function determining foliage orientation, θ is the solar elevation, λ is the effective foliage area volume density, which is summed over all crowns that the path intersects (l_t_). As with previous studies^11,12^, we assume a random foliage orientation of 0.5, l_t_ is calculated from the canopy thickness calculated above, and λ is a function of forest type and season. The probability is binarised across the hemispherical view (see^12^), which is then combined with the synthetic hemispheric image from T2R to create a synthetic hemispheric image within the forest, but also including terrain shading (Fig. 2a).

Two SVF variables are calculated:

1. *SVF-planar* represents the perspective of a horizontal/planar surface on the ground or snow surface; where the ratio of sky to canopy pixels in each 10° zenith angle ring is weighted by the ring’s surface area projected onto a horizontal flat surface.
2. *SVF-hemi* represents the perspective of a three-dimensional object on the ground, such as a plant, where the zenith rings are weighted by the surface area of each ring on the hemisphere.

DBT is calculated by first calculating the solar position (zenith and azimuth angle) using the parameterisation by NOAA^37^. The unique relative solar position is calculated at each model point at 2-minute resolution for the entire annual cycle, ensuring a temporally continuous solar track (spatially continuous in the synthetic hemispheric images). For each unique solar position, the DBT is the ratio of sky to canopy pixels in front of the solar disc projected into the image with an apparent diameter of approximately 0.53°^14^. In this study, we calculate the solar track at 2-minute resolution but average the output to save the final data at hourly resolution.

### Implementation and calibration

Using the 10 m forest mask, the T2R was run for every pixel in Switzerland, while the combination T2R + C2R was run within all the forest pixels, calculating hemispheric images for both leaf-on and leaf-off conditions. Since CanRad is a point-based model, images were calculated for the centre of each pixel but are assumed to represent the general canopy structure within each 10 m pixel.

CanRad has already been calibrated and validated for alpine Norway spruce forests in eastern Switzerland^18^. Additionally, the algorithms for calculating SVF and DBT from hemispheric images have been validated in^14,18^, therefore the focus of the calibration procedure for the SwissRad10 dataset was accurately representing the canopy structure within the synthetic hemispheric images in Swiss deciduous forests, and verifying performance in alpine needleleaf forests. To do this, we used hemispherical photographs taken during both leaf-on and leaf-off conditions in broadleaf forests and during leaf-off conditions in deciduous needleleaf forests.

The only information required by C2R in Eq. 1 that is not available or calculable over Switzerland is the foliage density, λ, which is used in C2R to also include woody elements of tree crowns. In the development of C2R, ref. ^18^ calculated this value by segmenting the canopy height model in their domain and using allometric equations to calculate a unique λ value for each individual tree crown. This method is impractical to apply over all of Switzerland’s forests. Instead, average λ values for each forest type/species were calculated in study areas using the tree crown segmentation method, and the forest type mix rate dataset from^28^ was used to interpolate λ values between forest types for each pixel in the canopy height model across the full model domain.

The λ value for evergreen Norway spruce forests in alpine regions was taken as the average value from the model domain used in^18^. In that study, they concluded that taking a domain-average value resulted in very similar model performance across the domain compared to tree-specific values. For larch forests in leaf-on conditions, we used the same λ value as for the evergreen forests.

The λ value for broadleaf forests during leaf-on conditions was calculated using the tree segmentation method in^18^ with allometric equations from^38^ for beech trees across the broadleaf domain shown in Fig. 1. The CHM was segmented across the broadleaf forest domain (Fig. 1), and a unique λ value was calculated for each individual tree. The domain-average was then taken to represent the leaf-on λ for all lowland broadleaf forests in Switzerland in a second model iteration, which was subsequently validated using hemispherical photographs.

Leaf-off λ values for deciduous species (both broadleaf and larch) were manually determined by iterating the model while comparing the output to leaf-off hemispheric photographs. Several iterations of the model were run for leaf-off scenarios, with the aim to reduce the statistical RMSD (root mean square difference) of the sky-view factor model output. Calculated synthetic hemispheric images were also visually compared with the photographs to ensure physically accurate representation of the canopy structure.

All λ values were then used with the mix rate data to calculate a λ value for each canopy pixel in the CHM. First, the forest type mix rate data was sub-sampled from 10 m to 1 m, the same resolution as the CHM. λ values were then applied directly to the CHM pixels, whereby 100% evergreen = λ_evergreen_ and 100% deciduous = λ_deciduous_, and values in between interpolated between the λ_evergreen_ and λ_deciduous_ values. Separate λ maps were calculated for leaf-on and leaf-off conditions. λ maps were then used in Eq. 1 to calculate light penetration probability and generate the synthetic hemispheric images across Switzerland.

## Data Records

**Fig. 3:**
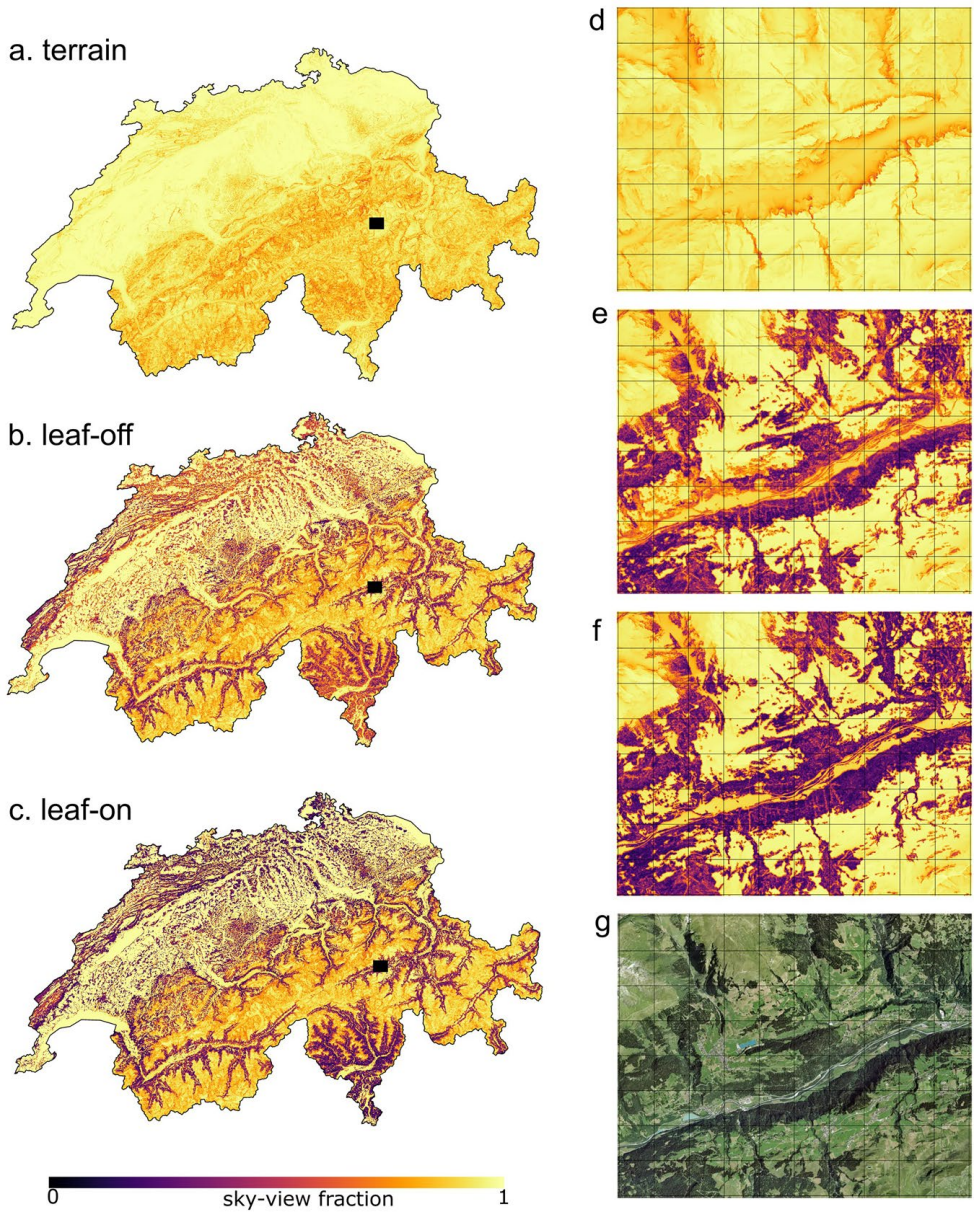
Calculated sky-view factor in the terrain, leaf-off and leaf-on forest datasets across Switzerland (**a**–**c**, respectively), and at an equivalent example 80 km^2^ area in central Switzerland (**e**,**f**; black region in **a**–**c**), aerial image showing forest structure (**g**). Gridlines in d–g are 1 km. Aerial image source: SWISSIMAGE 10 cm^44^.

The dataset^39^ generated in this study is available at https://www.doi.org/10.16904/envidat.544. The dataset includes both SVF and DBT variables within three sub-variables: terrain, leaf-on and leaf-off, shown in Fig. 3. Every 10 m pixel in Switzerland has a value in the terrain dataset, which includes only the influence of terrain shading and therefore also includes the light availability for the top of canopy within the forest mask. Only pixels inside the forest mask have data in the leaf-on and leaf-off layers, which represent light availability at the sub-canopy ground surface controlled by both terrain and surrounding forest structure. In total, 414,614,296 hemispheric images were calculated for the terrain dataset in every 10 m pixel within Switzerland, and 356,051,406 images were calculated for leaf-on and leaf-off conditions within the forested pixels, resulting in a total of 770,665,702 calculations.

The dataset consists of two file types. The SVF data is stored as three separate GeoTIFF files covering the entire model domain (Fig. 3). The DBT data is stored in NetCDF files, with separate files for each of the three sub-variables. The DBT data is further separated into daily files for ease of access. The data is saved at hourly resolution from 01.01.2020T00:00:00 to 31.12.2020T23:00:00 (a total of 8784 time steps), and each timestamp represents the beginning of the averaging period. All timestamps are CET (UTC + 1). Both DBT and SVF values are saved as 8-bit integers, representing percentages between 0–100 and should be scaled to fractions for estimating shortwave radiation transfer.

The dataset also includes the CHM and the forest mask used in the calculations (both in GeoTIFF format and covering the same spatial extent as the SVF data and daily DBT). All other datasets used in the calculations are already publicly available. The version of CanRad and associated scripts used to create the dataset are also included in the repository.

## Technical Validation

The validation of the dataset focuses on accurate representation of canopy structure in the synthetic hemispherical images as well as general representation of light availability across a heterogeneous landscape. Calculations of SVF and DBT from both hemispheric photographs and synthetic images are already validated in^14^ modelled shortwave radiation using calculations of SVF and DBT from hemispheric photographs, where we know canopy structure is correctly represented in the image. Comparison to measured shortwave radiation yielded a mean absolute error of only 19 Wm^−2^ and a root-mean square error of 28 Wm^−2^. These low values demonstrate that overall errors remain low as long as the canopy is represented correctly in the hemispheric images. Performance of T2R has also been verified in^14^ where it was used to mask snow-covered terrain in hemispherical photographs taken during winter (leaf-off) in alpine environments. The performance of C2R in representing alpine coniferous forests has already been validated in^18^. We extend the validation here to lowland deciduous broadleaf and mixed forests, as well as alpine larch (deciduous needleleaf) forests.

**Fig. 4:**
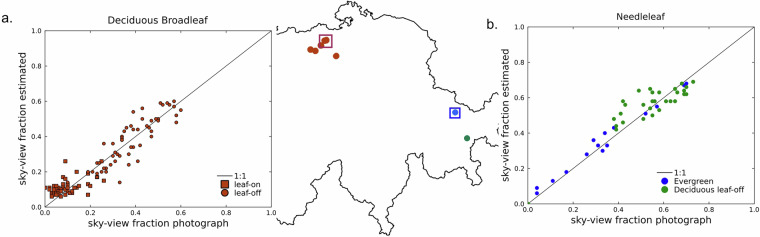
Comparison of calculated SVF from synthetic hemispheric images to those from hemispheric photographs taken in three different forest types in Switzerland; including both leaf-on and leaf-off values in deciduous broadleaf, leaf-off values for deciduous needleleaf and values for evergreen needleleaf. Exact locations of hemispherical photographs within inserts are shown in Fig. 5.

We validated SVF by (1) directly comparing output to values calculated from 90 hemispherical photographs taken in both leaf-on and leaf-off canopy conditions (Table 1) and (2) comparing model output to aerial imagery of Swiss forests taken in both conditions (Fig. 4). Validation was limited to locations where SVF was below 0.7 since this represents most forested environments and higher values typically correspond to locations within forest gaps or adjacent to forest edges^36^.

**Table 1:** RMSD (root mean square difference) between SVF calculated from hemispheric photographs and synthetic images calculated using T2R + C2R.

Forest type	SVF RMSD
Evergreen needleleaf	0.03
Deciduous needleleaf – leaf off	0.05
Deciduous broadleaf – leaf off	0.03
Deciduous broadleaf – leaf on	0.02

Root mean square difference values (Table 1) and scatterplots (Fig. 4) show the model suitably predicted sky-view factor throughout all dominant forest types in Switzerland. RMSD values were below 0.05 in all forest types, and variation around the 1:1 line in the scatter plots was relatively low. RMSD values were lowest in leaf-on deciduous broadleaf forests, but this is likely due to the typically low SVF values as a result of the denser foliage during leaf-on conditions. Deciduous needleleaf forests were not statistically evaluated for leaf-on conditions since corresponding hemispherical photographs were not available, but we expect the performance of the model to be similar to that in evergreen needleleaf forests due to a similar range in leaf-on tree crown density^40^.

**Fig. 5:**
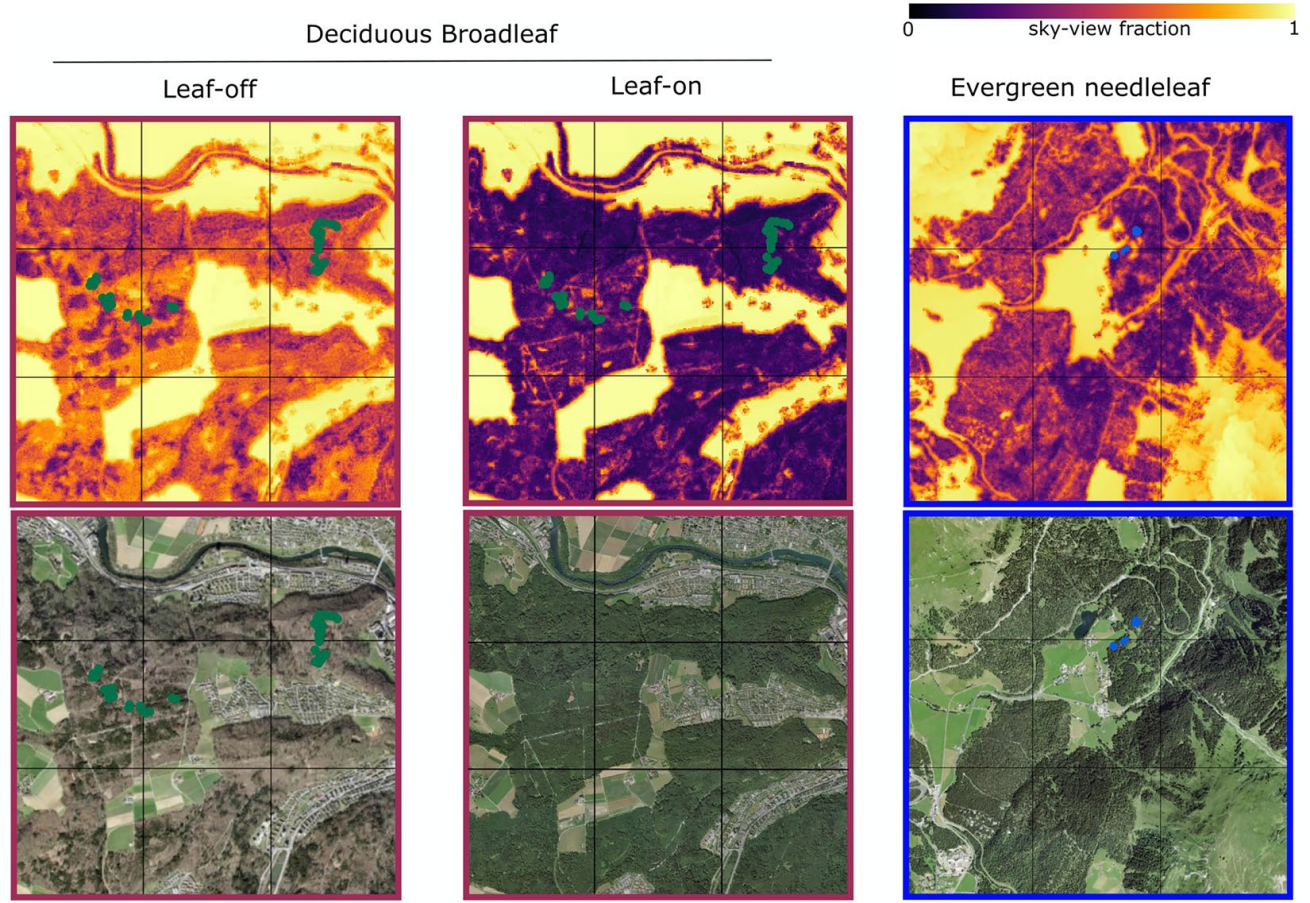
SVF maps and corresponding aerial images over two forested 9 km^2^ areas in Switzerland. The lowland deciduous broadleaf area is shown for both leaf-off and leaf-on canopy conditions, and the alpine evergreen needleleaf area has no seasonal change. Locations of 9 km^2^ grids within Switzerland are shown in the map inset in Fig. 4. Green and blue dots in the aerial images correspond to locations of hemispherical photographs used for validation in Fig. 4. Aerial image source: SWISSIMAGE 10 cm^44^.

Maps of predicted SVF in leaf-off conditions suitably replicate patterns seen in aerial images of leaf-off conditions, where areas of green forests (evergreen needleleaf) have lower SVF values compared to adjacent brown forests (deciduous broadleaf; Fig. 5). These patterns disappear in the leaf-on dataset, but paths and gaps in the forests are still clearly visible in the SVF maps, demonstrating the high spatial resolution and subsequent detail of the dataset (Fig. 5). While there is no seasonality to see in the SVF maps of the evergreen needleleaf forest, darker areas in the south of the aerial image correspond to lower SVF values in the model estimate maps, both indicating denser forest canopies.

**Fig. 6:**
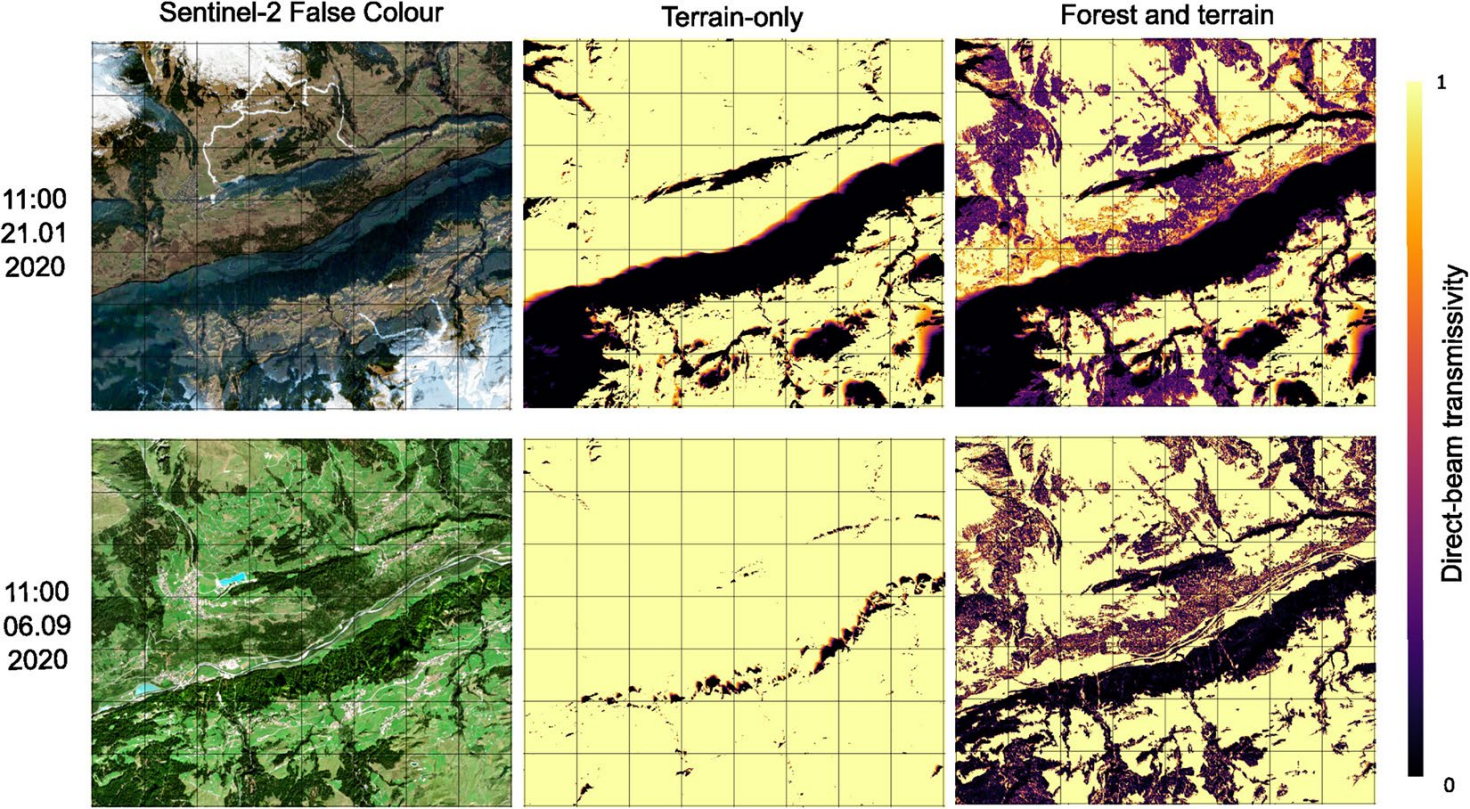
Comparison of Sentinel-2 image with the direct transmissivity model output for the same time period. The terrain-only map is included for both Sentinel-2 images, while the leaf-off data is compared to the Copernicus Sentinel-2 image on 21.01.2020 (top), and the leaf-on data is compared to the Copernicus Sentinel-2 image on 06.09.2020 (bottom).

DBT was validated at the landscape scale by comparing shadow maps from the model output with cloud-free Sentinel-2 false colour images taken during the same period, one in January (leaf-off) and one in September (leaf-on), both at 11am (Fig. 6). The main difference between the Sentinel-2 imagery is that it is a single snapshot, while the modelled data is averaged over the one-hour period. In spite of this difference, the modelled DBT data accurately capture the distribution and extent of the land surface shading caused by the complex topography, particularly in wintertime when a large portion of the area was shaded. In late summer (06.09.2020), local topographic shading at 11am covers a much smaller area, but is still well captured in the dataset.

## Usage Notes

### Calculation of solar radiation

DBT and SVF can be used to calculate incoming solar radiation at each time step *i* by multiplying the above-canopy incoming shortwave radiation separated into direct (SWR_dir_) and diffuse (SWR_dif_) components by the DBT and SVF, respectively:

2 $${{SWR}}_{i}={({SWR}}_{{dir},i}\ast {{DBT}}_{i})+({{SWR}}_{{dif},i}\ast {SVF})$$

Outside or above forests, the DBT and SVF values from the terrain dataset can be used to take into account the effect of the topographic terrain shading. To calculate below canopy radiation, DBT and SVF can be used from either the leaf off or leaf on dataset depending on leaf status.

In Eq. 2, above-canopy incoming shortwave radiation forcings could come from either weather station measurements, output from numerical weather prediction models such as COSMO in Switzerland, or reanalysis datasets such as ERA5. Where combined, the shortwave forcings can be split into the direct and diffuse components, using a partitioning scheme, for example^41^. If no such forcings are available, a maximum potential value can be used instead calculated by assuming an atmospheric transmissivity (τ_atm_) of 1 in:

3 $${{SWR}}_{i}={I}_{0}\ast \cos \left({\theta }_{i}\right)\ast {\tau }_{{atm}}$$

where *I*_*0*_ is the solar constant (1361 Wm^−2^) and *θ* is the solar zenith angle at time step *i*.

### Periods of green-up and leaf senescence

DBT for leaf-on and leaf-off scenarios were calculated across the entire annual cycle and SVF was calculated for each of the two scenarios. It is therefore up to the user’s discretion how to combine the two datasets when wanting SVF and DBT to vary across the annual cycle including transition periods during green-up and leaf senescence. This either requires site-specific data on vegetation phenology, or a synergy of the nationwide dataset with, for example, monthly satellite-derived NDVI data.

### Representativeness of canopy structure data

The year 2020 was calculated to include 29 February in the final dataset, but the data is transferable to other years since the data is not dependent on meteorological conditions and the solar track does not significantly change between annual cycles. Importantly however, the canopy height model is based on lidar data collected across Switzerland between 2012 and 2022. The forest mix rate dataset was calculated in 2020. Light availability calculations are therefore representative of forests at time of acquisition on input datasets.

Additionally, the mix rate dataset is derived from satellite datasets using machine learning methods, and therefore likely to have small-scale errors and inconsistencies. Although we supplement this dataset with the Copernicus dominant leaf type information, users of the SwissRad10 dataset who are interested in smaller areas should check how well the mix rate dataset represents their region.

### Data exclusion

1. Buildings were not included as a dataset for calculating the synthetic hemispheric images, therefore all built-up and urban areas are treated as open terrain.
2. The DTM-5m, CHM and mix rate datasets are only available up to the Swiss border. An under-estimation of light availability in forests within 50–100 m inside the border is to be expected where canopy information was not available in all directions. We do not expect large errors in estimation of terrain-only light availability due to the use of the DTM-50m data out to 25 km beyond the Swiss border. There are likely fewer edge issues along areas of the border that are designated by a topographic break (e.g. southern and eastern Switzerland), compared to the northern and western borders.

### Early uses

An earlier iteration of the leaf-off dataset has been used as input for the operational snow hydrology forecasting service, which averaged the dataset to 250 m resolution and used this for the canopy structure variables in their snow model in combination with COSMO predicted incoming shortwave radiation following^20,42^. An additional 100 m resolution product is included in^43^. A 20 m version of the leaf-on output has been interpolated to 10 m and used with maximum potential incoming shortwave radiation in a statistical model to calculate microclimate maps over Switzerland^5^.

## Data Availability

The dataset is publicly available on EnviDat (https://www.doi.org/10.16904/envidat.544) under the Creative Commons Zero (CC0 1.0) license.

## Code availability

The version of CanRad used in the data calculation is available with the data repository, along with the additional scripts used to generate the stored data. The most recent version of the model is available on github as the julia package: https://github.com/c-webster/CanRad.jl.
